# Genotypic characterization of gentamicin and cephalosporin resistant *Escherichia coli* isolates from blood cultures in a Norwegian university hospital 2011–2015

**DOI:** 10.1186/s13756-017-0280-2

**Published:** 2017-11-29

**Authors:** Øyvind Andreas Fladberg, Silje Bakken Jørgensen, Hege Vangstein Aamot

**Affiliations:** 1 Department of Microbiology and Infection Control, Akershus University Hospital, Lørenskog, Norway; 2 Department of Medical Biochemistry and Microbiology, Uppsala University, Uppsala, Sweden; 3 Department of Clinical Molecular Biology (EpiGen), Division of Medicine, Akershus University Hospital and University of Oslo, Lørenskog, Norway

**Keywords:** ESBL, CTX-M, AmpC, MLVA, Bacteraemia, *E. coli*, Antibiotic resistance, Genotyping, Surveillance

## Abstract

**Background:**

Cephalosporin resistance in clinical *E. coli* isolates is increasing internationally. The increase has been caused by virulent and often multidrug-resistant clones, especially the extended spectrum β-lactamase (ESBL) producing *E. coli* clone O25b-ST131.

**Methods:**

In Norway, recommended empirical treatment of sepsis consists of gentamicin and penicillin combined, or a broad-spectrum cephalosporin. To investigate if increased gentamicin and cephalosporins resistance rates in our hospital could be caused by specific clones, we conducted a retrospective study on *E. coli* blood culture isolates from 2011 through 2015. All *E. coli* isolates non-susceptible to gentamicin and/or third-generation cephalosporins were genotyped using multiple-locus variable-number of tandem repeat analysis (MLVA) and compared with antibiotic susceptible isolates. The frequency of the most common genes causing ESBL production (*bla*_CTX-M_, *bla*_ampC_) was examined by Real-Time PCR.

**Results:**

A total of 158 cephalosporin and/or gentamicin resistant and 97 control isolates were differentiated into 126 unique MLVA types. Of these, 31% of the isolates belonged to a major MLVA cluster consisting of 41% of the gentamicin resistant and 35% of the cephalosporin resistant isolates. The majority (65/80 isolates) of this MLVA cluster contained MLVA types associated with the *E. coli* O25b-ST131 clone. Genes encoding CTX-M enzyme phylogroups 1 and 9 occurred in 65% and 19% of cephalosporin resistant isolates, respectively, whereas *bla*_ampC-CIT_ was identified in 3%.

**Conclusion:**

No local *E. coli* bacteraemia clone was identified. Antibiotic resistance was dispersed over a variety of genotypes. However, association with the international *E. coli* O25b-ST131 clone was frequent and may be an important driver behind increased resistance rates. Monitoring and preventing dissemination of these resistant clones are important for continued optimal treatment.

**Electronic supplementary material:**

The online version of this article (10.1186/s13756-017-0280-2) contains supplementary material, which is available to authorized users.

## Background

*Escherichia coli (E. coli)* is the most common cause of bacteraemia [1–3]. In Norway, national recommendations for empirical treatment of sepsis are gentamicin and penicillin combined, or a broad-spectrum cephalosporin (https://helsedirektoratet.no/retningslinjer/antibiotika-i-sykehus, accessed 22. June 2017). According to the Norwegian surveillance programme for antimicrobial resistance in human pathogens (NORM), resistance to gentamicin in *E. coli* from blood cultures more than tripled the last decade from 2.0% in 2005 to 6.4% in 2015 [3, 4]. The prevalence of extended β-lactamases (ESBLs) increased from 0.5 to 6.5% in the same period. In 2015, approximately 40% of the gentamicin resistant isolates also produced ESBLs, but with large variations between different Norwegian hospitals [3].

ESBLs are the leading cause of resistance to broad-spectrum cephalosporins in *E. coli*, together with AmpC-enzymes and enzymes also conferring resistance to carbapenems [5]. The most common ESBLs are the CTX-M enzyme family [2]. The family is divided into six phylogroups, where CTX-M phylogroup 1 is the most frequent. The dominance of CTX-M can be attributed to the successful *E. coli* clone O25b-ST131 carrying CTX-M-15, belonging to CTX-M-1 phylogroup, [6, 7] and the association with conjugative plasmids [8]. Among the plasmid-borne AmpC β-lactamases, there are six groups of AmpC genes of which the *bla*_CMY-2_ is the most common [2].

Use of molecular typing methods is essential in both surveillance and outbreak investigations as well as in tracking of evolutionary trends of microbes in both the community and healthcare settings. Pulsed field gel electrophoresis (PFGE), the previous “gold standard” typing method, has become outdated due to a time-consuming and labour-intensive protocol. Multilocus sequence typing (MLST) is widely used and has an advantage as the generated data can easily be shared between laboratories. However, this method does not give sufficient resolution for local outbreak investigations [9]. Whole genome sequencing is on the rise, but is still sub-optimal for small-scale surveillance and outbreak investigations in real-time. Multiple-locus variable-number of tandem repeat analysis (MLVA) has been performed to assess clonal relatedness between *E. coli* isolates. It has been reported as a rapid, accurate and cost-effective genotyping tool suitable as a “first-line-of-defense” method for epidemiological surveillance of multidrug-resistant *E. coli* [9–12]. In previous studies, *E. coli* O25b-ST131 has been shown to be associated with distinct MLVA types [9, 11–13].

Managing antibiotic resistance is a global issue and in order to limit dissemination effectively, monitoring genotypes and resistance mechanisms is vital [14]. To investigate if gentamicin resistance in *E. coli* bacteraemia was linked to one or several clones co-resistant to cephalosporins, we performed MLVA on all gentamicin and/or third-generation cephalosporin resistant *E. coli* from diagnostic blood cultures at our hospital from 2011 to 2015. MLVA analysis was chosen due to technological availability and low cost. In addition, the analysis is easy to perform and the data generated is simple to handle and interpret. We also wanted to investigate the prevalence of genes causing ESBL production.

## Methods

### Study design and setting

This retrospective cross-sectional study was performed at Akershus University Hospital (Ahus), Lørenskog, Norway. Ahus delivers public health services to ~ 500,000 people and is the largest acute care hospital in Norway.

### Culturing and antibiotic susceptibility testing

**Fig. 1:**
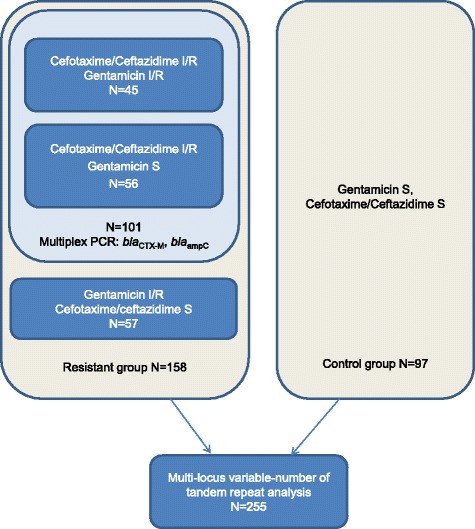
Sample flow chart of the selected *E. coli* isolates from blood cultures. S = susceptible, I = intermediate, R = resistant

All gentamicin non-susceptible (*N* = 102) and/or third-generation cephalosporin (ceftazidime and/or cefotaxime) non-susceptible (*N* = 101) *E. coli* from diagnostic blood cultures at Ahus from 2011 to 2015 were included (one isolate per patient per year). Co-resistance to gentamicin and cephalosporins were present in 45 isolates, hence a total of 158 non-susceptible isolates were included. Additionally, gentamicin sensitive and third-generation cephalosporin sensitive isolates (*n* = 97, ~ 20/year, one isolate per patient), were included as controls. A sample flow chart is presented in Fig. 1.

The samples were cultured in the BD BACTEC FX blood culture system (BD Diagnostics, Heidelberg, Germany) and identification was performed by conventional fermentation reactions or mass spectrometry (MALDI-TOF, Bruker Daltonics, Bremen, Germany). Phenotypic antibiotic susceptibility testing was determined using disk diffusion tests according to the Nordic committee on Antimicrobial Susceptibility Testing guidelines (www.NordicAST.org). ESBL phenotypes were detected by the disk approximation test [15], and AmpC phenotypes were detected by AmpC MIC strips (Etest, BioMérieux, Marcy l’Etolie, France and Mic Test Strip, Liofilchem, Roseto degli Abruzzi, Italy).

Intermediate and resistant (non-susceptible) phenotypes were combined in further analysis. Hence, in the following, all non-susceptible isolates, i.e. intermediate or resistant according to EUCAST (European Committee on Antimicrobial Susceptibility Testing) break points (www.eucast.org), are referred to as resistant.

### Multiple-locus variable-number tandem repeat analysis (MLVA)

All isolates from blood cultures were stored at −80 °C. DNA was extracted by boil lysis before standardisation [10 ng/μL]. MLVA was performed according to published protocol by Linstedt and co-workers using seven alleles [16] with modification by Løbersli and co-workers [17]. Fragment analysis was performed by capillary electrophoresis on the 3130xl Genetic Analyzer (Applied Biosystems, Sint-Martens-Letem, Belgium). Determination of MLVA types and creation of minimum spanning trees were performed using BioNumerics software (Applied Biosystems 7.1). No PCR product was denoted as “-2”, whereas no repeats was denoted as “0”. Otherwise, the allele numbers reflect the number of repeats found in each locus. The MLVA types were numbered consecutively and designations are unique to this work (Additional file 1: Table S1). Dendrograms were constructed using categorical coefficients and the Ward algorithm and a standard minimum spanning tree was generated using categorical coefficients together with the single and double locus variance priority rules.

### CTX-M Real-Time PCR

CTX-M Real-Time PCR was performed according to published protocol with minor modifications [18]. The original quadplex Real-Time PCR was divided into a duplex for CTX-M enzyme phylogroups 1 and 9, and a singleplex for CTX-M phylogroup 2. Due to low occurrence of CTX-M variants detected exclusively with the degenerate probe in the population, this probe was excluded. Analysis was performed on ABI PRISM 7900HT sequence detection system (Applied Biosystems, Foster City, CA, USA) with two detectors (FAM and VIC) in addition to ROX as passive reference.

### AmpC Real-Time PCR

Detection of plasmid-borne AmpC was performed on phenotypically AmpC positive isolates according to published protocol with minor modifications [19]. The modified protocol was limited to detection of the most frequent genes previously described in Norway [20], *bla*_DHA_ and a combination of *bla*_CIT_ and *bla*_CMY_.

## Results

From 2011 to 2015, 1475 *E. coli* isolates were derived from blood cultures. Of these, 102 (6.9%) were resistant to gentamicin and 101 (6.8%) were resistant to third-generation cephalosporins. 45 (3.1%) of the isolates were co-resistant to both drugs. Of the 101 third-generation cephalosporins resistant isolates, 85 (84%) were phenotypically positive for ESBL and 13 (13%) positive for the AmpC phenotype test.

### MLVA

**Fig. 2:**
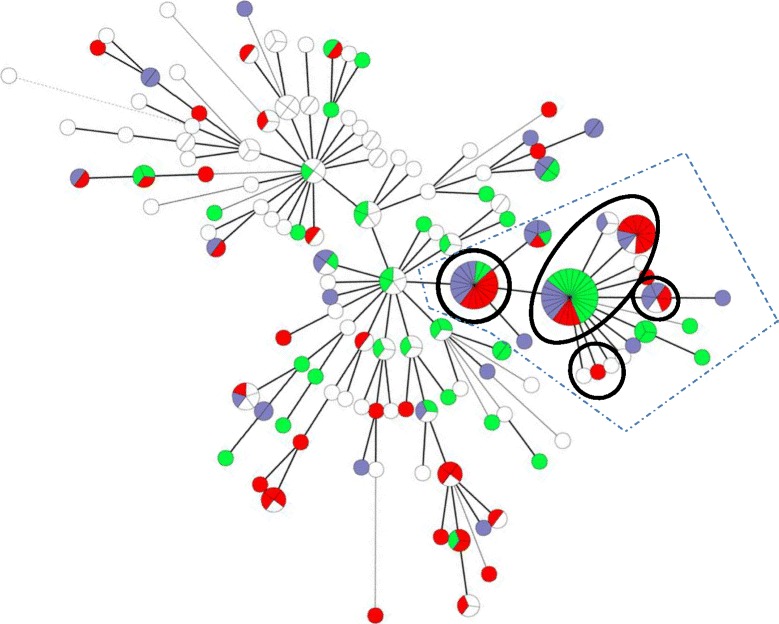
Minimum spanning tree based on the 126 MLVA types (MTs) found in 256 *E. coli* bacteraemia isolates from 2011 to 2015. The isolates are coloured by phenotypic resistance. White: cephalosporin S and gentamicin S, *green* cephalosporin R and gentamicin S, *red*: cephalosporin S and gentamicin R, purple: cephalosporin R and gentamicin R. One disk represents one MT. Disk size indicates the number of isolates in the MT. The length of branches specifies how many Variable Number of Tandem Repeats (VNTR) loci are different, where the shortest branches indicate 1 VNTR loci separating MTs. This only applies to adjacent MLVA disks. Encircled MTs are associated with the international *E. coli* O25b-ST131 clone. *Dotted line* indicates the major MLVA cluster

**Fig. 3:**
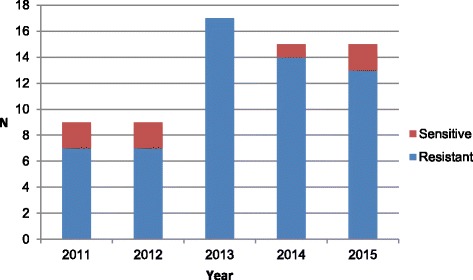
Number of *E. coli* isolates with MLVA type associated with *E. coli* O25b-ST131 clone per year of inclusion

Distribution of antibiotic resistance profiles by MLVA types (MTs) is presented in Fig. 2. The 158 antibiotic resistant isolates and 97 controls were differentiated into 126 unique MLVA types. A total of 65 (25%) of the isolates had MLVA types previously associated with O25b-ST131 (MT: 6, −2, −2, 14, 3, X, 5) [9, 11, 13] of which 7 (3%) were antibiotic susceptible controls. Of the gentamicin resistant isolates, 42/102 (41%) belonged to this cluster, as did 35/101 (35%) of the cephalosporin resistant isolates. For overview of all MLVA types see Additional file 1: Table S1. The occurrence of the ST131 associated MLVA types varied from seven to 17 per year (Fig 3) among the antibiotic resistant isolates and the number increased during the study period.

There was no exclusive association between MLVA type and phenotypic resistance, but resistance to third-generation cephalosporins dominated the largest MLVA type (MT 79, 20/24 isolates, Fig 2).

### CTX-M and AmpC prevalence

A total of 85/101 (84%) of the isolates resistant to third-generation cephalosporin were positive for CTX-M genes corresponding to the 85 isolates positive for ESBL phenotype. CTX-M phylogroup 1 was most common with 66/101 isolates. CTX-M phylogroup 9 was identified in 18/101 isolates. In addition, one isolate was positive for both phylogroups 1 and 9. The remaining 16 isolates were negative for *bla*_CTX-M_. None of the isolates were positive for CTX-M phylogroup 2.

Of the 13/101 third-generation cephalosporin resistant isolates that were phenotypically positive for the AmpC-test, three were positive for plasmid-borne AmpC genes, all of which were *bla*_CIT/CMY2._ No isolate carried the combination of *bla*_CTX-M_ and *bla*_CIT/CMY2_ genes. However, two isolates that were phenotypically AmpC (presumptively identified as chromosomal-encoded AmpC) also contained genes from CTX-M phylogroup 1. With the combination of CTX-M Real-Time PCR and phenotypic AmpC, we identified the cause of resistance in 96/101 (95%) of the third-generation cephalosporin resistant isolates.

## Discussion

The 7-locus MLVA identified several frequent clones in blood cultures at our hospital. They were found both in community-onset bacteremia and in hospital-acquired infections from different hospital wards and spread over a large time span. Hence, MLVA did not identify a hospital specific clone. However, MLVA did show that gentamicin and third-generation cephalosporin resistant isolates are more similar than antibiotic sensitive *E. coli*, i.e. they are more likely to belong to certain MLVA types. MLVA identified a large cluster that almost exclusively consisted of antibiotic resistant isolates. In this cluster, gentamicin resistance was more frequent than cephalosporin resistance or resistance to both drugs. An association between the O25b-ST131 clone and MTs (6, −2, −2, 14, 3, X, 5) has previously been described [6, 8, 11, 21] and the number of isolates with these MLVA types increased during the study period (Fig 3). Half (9/18) of the MLVA types in this cluster, containing 65/80 of the isolates, are associated with the pandemic O25b-ST131 clone, illustrating the considerable disease contribution from O25b-ST131 in an otherwise heterogenic population.

Gentamicin resistance was even more frequent than cephalosporin resistance in the O25b-ST131 associated MLVA types. Genes conferring aminoglycoside resistance and *bla*_CTX-M_ may occur on the same plasmids [22]. Gentamicin resistance can also be caused by adaptive mechanisms, such as membrane protein changes and regulation of genes involved in the anaerobic respiratory pathway.

Globally, the dominating cause of ESBL is the CTX-M enzyme family [2, 23]. The prevalence of the most common ESBL genes was as anticipated with CTX-M phylogroup 1 as the most frequent (65%) followed by CTX-M phylogroup 9 (19%) which is in accordance with previously published findings [3, 24]. CTX-M was highly prevalent (84%) among third-generation cephalosporin resistant isolates in this study. The absence of CTX-M phylogroup 2 was as expected. The Norwegian National Advisory Unit of Antimicrobial Resistance did not detect this CTX-M group in blood culture isolates from 2000 through 2014 [3] and a Swedish study reported that only 2 of 1003 (<0.002%) of urinary tract infection derived *E. coli* isolates were CTX-M phylogroup 2 [24]. With the combination of methods employed in this study we determined the cause of resistance in 95% of the isolates with third-generation cephalosporin resistance. The resistance mechanisms in the remaining isolates can possibly be explained by loss of porins resulting in lower permeability, loss of the resistance plasmid [25] or other CTX-M phylogroups.

Surveillance of plasmid-borne CTX-M and AmpC in combination with interventions to limit their dissemination can ensure longevity of the current recommendations for empirical sepsis treatment. Adequate, real-time surveillance should be employed in hospitals to identify outbreaks of antibiotic resistant and virulent strains in order to stop dissemination as early as possible. Parallel analysis of genotype and detection of resistance genes by Real-Time PCR is a pragmatic way of ensuring early outbreak detection. Using MLVA with only 3 VNTRs (CVN001, CVN004, CVN014) has been suggested to be adequate for epidemiological screening of ESBL-producing *E.coli* [9]. In our study, using the 3 VNTR MLVA would have given 84 MLVA types compared to the 126 MLVA types with all 7 VNTRs reducing the number of MLVA types with 33%. However, the differentiation of O25b-ST131 associated MLVA types was identical between 3 and 7 VNTR MLVA resulting in 9 MLVA types with both methods. As O25b-ST131 associated MLVA types comprised several major MLVA types in our study, complementary techniques may be necessary to differentiate among O25b-ST131 isolates in local outbreak investigations, as previously reported by Helldal and co-workers [9].

There are limitations to our study. Only a random selection of antibiotic sensitive *E. coli* bacteraemia isolates was included as controls for MLVA typing (~ 20 isolates per year of inclusion). Therefore, the specific MLVA types they represent may be biased. In general, however, they show more heterogeneity than the antibiotic resistant *E. coli* isolates. The Real-Time PCR for detection of CTX-M phylogroups was designed only to detect the three most common phylogroups (1, 2 and 9) leaving a possibility for false negatives. However, the prevalence of other phylogroups is low in Norway [3] and we detected the cause of resistance in 95% of the cephalosporin resistant isolates. The number of phenotypic AmpC positive *E. coli* to include in the study was scarce. Yet, over the 5 years included in the study, a total of 1475 *E. coli* was identified and they were all potential candidates for inclusion. The performed Real-Time PCR detected the two most common AmpC groups constituting 99% of previously reported cases [20]. Therefore, in the case of isolates negative for plasmid-borne AmpC, chromosome-encoded AmpC was presumptively identified. Extrapolation of data from this study should consider that isolates were extracted from individuals in a specific geographic region.

## Conclusion

No local *E. coli* bacteraemia clone was identified. However, a large cluster of related MLVA types, comprising 25% of the isolates, was associated with the international *E. coli* O25b-ST131 clone suggesting that this clone is a major driver behind the increase in cephalosporin and gentamicin resistance in an otherwise heterogenic population. Resistance to third-generation cephalosporins was identified in 95% of the isolates with presence of genes coding for CTX-M phylogroup 1 as the most frequent cause of resistance. Monitoring and preventing dissemination of these resistant clones are important for continued optimal treatment.

## Additional file

Additional file 1: Overview of all 255 *E. coli* isolates from bacteremia with regards to antibiotic resistance profiles, MLVA type, and *bla*_CTX-M_ and *bla*_ampC_. (XLS 82 kb)

## Abbreviations

*E. coli*: *Escherichia coli*
ESBL: Extended spectrum β-lactamase
MLVA: Multiple-locus variable-number of tandem repeat analysis
MT: MLVA type
VNTR: Variable number of tandem repeat
